# Healthy vending contracts: Do localized policy approaches improve the nutrition environment in publicly funded recreation and sport facilities?

**DOI:** 10.1016/j.pmedr.2019.100967

**Published:** 2019-08-07

**Authors:** Cassandra Lane, Patti-Jean Naylor, Dona Tomlin, Sara Kirk, Rhona Hanning, Louise Masse, Dana Lee Olstad, Rachel Prowse, Susan Caswell, Sherry Jarvis, Todd Milford, Kim Raine

**Affiliations:** a School of Medicine and Public Health, Faculty of Health and Medicine, University of Newcastle, 1 University Drive, Callaghan, NSW 2308, Australia; b Institute of Applied Physical Activity and Health Research, School of Exercise Science, Physical and Health Education, University of Victoria, PO Box 3015, STN CSC, Victoria V8W 3P1, BC, Canada; c Healthy Populations Institute, Dalhousie University, PO Box 15000, Halifax B3H 4R2, NS, Canada; d School of Public Health & Health Systems, Faculty of Applied Health Sciences, University of Waterloo, 200 University Ave. W., LNH 3117, Waterloo N2L 3G1, ON, Canada; e BC Children's Hospital Research Institute, School of Population and Public Health, University of British Columbia, 2206 East Mall, Vancouver V6T 1Z3, BC, Canada; f Department of Community Health Sciences, Cumming School of Medicine, University of Calgary, 3280 Hospital Drive NW, Calgary T2N 4Z6, AB, Canada; g Centre for Health and Nutrition, School of Public Health, University of Alberta, 4-077 Edmonton Clinic Health Academy, 11405 – 87 Ave., Edmonton T6G 1C9, AB, Canada

**Keywords:** Public facilities, Nutrition policy, Contracts, Food dispensers, Automatic, Pediatric obesity

## Abstract

This study explored the influence of healthy vending contracts (HVC) on the nutritional quality of vending machine products in 46 Canadian publicly funded recreation and sport facilities. A quasi-experimental comparison design was used to examine the difference in nutritional quality of snack and beverage vending machine products at baseline (December 2015–May 2016) and 18-month follow-up. Staff Surveys assessed facility contract type (HVC or conventional) and vending machine audits identified product nutritional quality. Products were categorized by provincial guidelines as Do Not Sell (DNS), Sell Sometimes (SS) or Sell Most (SM). ANOVA compared categories cross-sectionally (HVC vs conventional) and repeated measures ANOVA compared them longitudinally (HVC-HVC, vs conventional-conventional and conventional-HVC).

Approximately one quarter of contracts (24% beverage and 28% snack) had health stipulations at baseline or follow-up. Cross-sectionally, facilities with HVC at any time period had significantly lower percentage DNS (beverage: 56% vs 73%, *p* = 0.001; snack: 55% vs 85%, *p* < 0.001), higher SS (beverage: 24% vs 14%, *p* = 0.003; snack: 35% vs 12%, *p* < 0.001) and higher SM Products (beverage: 21% vs 13%, *p* = 0.030; snack: 10% vs 3%, *p* < 0.003). Longitudinally, facilities with consistent HVC or that changed to HVC showed greater decreases in DNS products over time (*p* < 0.050).

Although less healthy products were still highly prevalent, facilities with HVC or that changed to HVC had fewer unhealthy products available in their vending machines over time compared to those without HVCs. Healthy vending contracts appear to be an effective change strategy.

## Highlights

• Healthy vending contracts are associated with healthier vending product profiles.
• Healthy vending contracts improved the ‘healthiness’ of the products sold in vending machines.
• Healthy vending contracts appear to improve the food environment of recreational and sport facilities.
• Unhealthy foods remain prevalent in recreational and sport facilities even when healthy vending contracts are in place.

## 1 Introduction

A majority of Canadian children are not consuming healthy diets (Rao et al., 2017). This is concerning considering the association between unhealthy food consumption and increasing rates of childhood obesity (World Health Organization, 2016; Public Health Agency of Canada, 2011); the health repercussions of which persist throughout the life course and place a substantial (and often inequitable) burden on individuals, broader society and economies worldwide (Vallgårda, 2018). While the causative pathways to childhood obesity are complex, public health experts have increasingly recognized the contribution of food environments (World Health Organization, 2016; Roberto et al., 2015; Raine et al., 2018) where unhealthy foods are readily available and accessible to children (World Health Organization, 2016).

In Canada, unhealthy food environments are commonplace in public facilities (Raine et al., 2018), including recreation and sport centres (Thomas and Irwin, 2010; Naylor et al., 2010; Chaumette et al., 2009; Olstad et al., 2019) which are frequently visited by children (Thomas and Irwin, 2010; Naylor et al., 2010; Olstad et al., 2011a). This not only contributes to the childhood obesity epidemic, but also contradicts their responsibility to provide conditions that are conducive to the good health of citizens (Raine et al., 2018), and may undermine efforts to address unhealthy food environments in other settings (Olstad et al., 2011a; Engler-Stringer et al., 2014; Olstad et al., 2013).

Food and beverage policy has strong potential to improve unhealthy food environments (Roberto et al., 2015; Raine et al., 2018; Ashe et al., 2007; Schwartz et al., 2017). In addition, evidence indicates an increased availability, sale and intake of healthier products in Canadian public facilities where such policies are in place (Raine et al., 2018). Unfortunately food and beverage policies in these settings remain minimal (Raine et al., 2018). Some Canadian provinces have developed guidelines that set nutrition standards for foods and beverages available in publicly funded recreation and sport facilities, yet none have achieved the specified nutrition standards (Olstad et al., 2011a; Olstad et al., 2013; Naylor et al., 2015) and numerous barriers to implementation have been cited (Olstad et al., 2013; Naylor et al., 2015; Olstad et al., 2011b). This suggests that further policy action may be required (World Health Organization, 2016; Public Health Agency of Canada, 2011) and research is needed to distinguish where to target policy efforts (Vallgårda, 2018).

Incorporating health stipulations into vending machine contracts (i.e., developing a healthy vending contract [HVC]) may be a viable facility-level policy option for several reasons. First, contracts are a direct and legal means through which modifications to vending machine products may be promoted (Ashe et al., 2007). The food and beverage industry historically used vending contracts to promote the sale of unhealthy consumables (Nestle, 2000). HVCs may alternatively promote the availability and normalization of healthy foods and beverages in vending machines. Second, substantial evidence has linked localized healthy vending machine initiatives with significant improvements in the nutritional quality of vending – i.e., increased healthy options and/or decreased unhealthy options (Olstad et al., 2013; Naylor et al., 2015; Naylor et al., 2010b; Brooks et al., 2017; Boelsen-Robinson et al., 2017; Lillehoj et al., 2015; Mason, 2014; van Hulst et al., 2013; Bell et al., 2013). Third, a localized intervention designed to support provincial nutrition guideline implementation in British Columbia recreation facilities experienced more substantial improvements where HVCs were in place (Naylor et al., 2015; Naylor et al., 2010b). Lastly, the development of HVCs is likely a necessary step to ensure the full implementation of voluntary provincial nutrition guidelines. Healthy food service contracts have been specifically reported as a feasible means of implementing nutrition guidelines in recreation facilities (Olstad et al., 2011a), and conventional vending contracts (i.e., those without any health stipulations) have been cited as a barrier to the implementation of higher level food and beverage policies (Olstad et al., 2013; Naylor et al., 2015; Bell et al., 2013).

Expanding the evidence-base on healthy food procurement policies in Canada (Raine et al., 2018) and promoting health in children's sport facilities internationally have been recommended (World Health Organization, 2016). However, there remains a shortage of evidence about the efficacy of policy interventions in this setting. More specifically, we need to know more about the impact of HVCs, a form of food and beverage policy, on the unhealthy food and beverage environments within publicly funded recreation and sport facilities. Thus, the purpose of this study was to fill an important evidence gap by examining the effect of HVCs on food and beverages provided in Canadian publicly funded recreation and sport facilities. To achieve this, we examined the differences between the nutritional quality of products sold in snack and beverage vending machines in facilities with and without HVCs at one point in time (cross-sectionally), and over time (longitudinally) to establish whether there was a plausible causal pathway.

## 2 Methods

### 2.1 Research design

A non-equivalent quasi-experimental comparison design was used to address the research objectives. The study was conducted using data from the broader Eat, Play, Live (EPL) trial: a natural experiment with an embedded randomized comparison trial examining the impact of provincial nutrition guidelines and capacity-building on food environments in publicly funded recreation and sport facilities (Olstad et al., 2019). Data used for this sub-study were from the vending and survey data collected at baseline (T1; December 2015–May 2016) and 18-month follow-up (T2).

### 2.2 Ethics statement

Ethical approval for this research was provided by the Research Ethics Boards at the University of Victoria and University of British Columbia (Harmonized # BC 15-196), the University of Alberta (#Pro00058096), the University of Waterloo (#20913) and Dalhousie University (#2015-3637).

### 2.3 Participation

Forty-nine publicly funded recreation and sport facilities across four Canadian provinces; British Columbia (n = 14), Alberta (n = 11), Ontario (n = 17), and Nova Scotia (n = 7) participated in the EPL trial. As previously outlined (Olstad et al., 2019), they were recruited via notices distributed by provincial Recreation and Parks Associations (or similar organizations) on websites, at meetings and via emails to their membership lists. Two-hundred and eighty-six facilities expressed interest and were then contacted by the research team. Only 145/286 returned phone calls/emails and 70 were ineligible because they were currently or recently (within the last five years) involved in changing their food environment, did not provide any food services, or did not provide recreation and sport programming. Of the remaining 75 facilities, 11 lacked staff capacity, two were not interested in the research component, one feared randomization into the comparison group and one revenue loss, and 11 did not provide a reason. Forty-nine (65%) of facilities who returned calls and were eligible participated, and only those with vending machines (n = 46) were included in this sub-study.

### 2.4 Data collection and treatment

Surveys to assess contract status (condition) were administered and standardized vending product nutrition quality audits (Naylor et al., 2010b) were conducted by the research team at T1 and T2. Specific data collection details are described following.

#### 2.4.1 Vending contract status

Staff representatives completed questionnaires administered by the research team via e-mail or telephone when e-mail response was slow. Questions asked if a vending contract existed, what type of product they targeted (i.e., snack, beverage, or all vending contracts which covered both snack and beverage) and if they had a health stipulation (at a minimum mentioned health). Other health stipulations included specifying the proportion of products that must be ‘healthy’ or ‘unhealthy’ and whether someone was assigned to monitor adherence to the healthfulness of vending products. Survey responses were used to classify contracts; if they included health stipulations they were classified as healthy (HVC) and if they did not they were conventional.

#### 2.4.2 Vending machine audits

We employed a four-step standardized vending machine audit procedure with previously established inter-rater reliability (test-retest and inter-rater reliability ≥0.88) (Naylor et al., 2010b). Step 1. Trained research assistants recorded the number, type (snack or beverage) and location of all vending machines at participating facilities and assigned them unique codes to aid random selection. Step 2. A remote independent researcher randomly selected a maximum of two snack and two beverage machines for audit. Step 3. A standardized audit form detailing product brand, variety/type, size, flavour and price was used to generate a product list for each vending machine. Step 4. Product information was entered into an automated provincial nutrient profiling system for packaged foods (the British Columbia Brand Name Food List; Dietitian Services) which categorizes foods based on dietitian verified product information and the British Columbia guidelines (Province of British Columbia, 2014).

Although provincial guideline nutrient profiling systems differ across jurisdictions, they offer moderate to good agreement on product nutritional quality and similar policy outcomes (Olstad et al., 2015). The British Columbia guidelines were used to harmonize outcomes for cross-province analysis. The three categories within these guidelines are currently described as: Sell Most (SM; healthiest options due to greater nutritional value and lower in sodium, sugar and fat– e.g., packaged nuts, mini rice cakes and water), Sell Sometimes (SS; contains essential nutrients but higher in sodium, sugar and fat than SM– e.g., soft baked oatmeal cookies and fruit juice with no added sugar), and Do Not Sell (DNS; high energy density and high in sodium, sugar and/or fats– e.g., cereal bars with sugar as the first ingredient, nacho cheese tortilla chips and teriyaki beef jerky). Further information about the nutrient categorization system can be found at (www.healthlinkbc.ca). The final counts for each category were converted into percentages to account for variability in the number of slots between vending machines.

### 2.5 Data analysis

Statistical Package for the Social Sciences (SPSS Version 22.0 [IBM Inc]) was used for all analysis.

#### 2.5.1 Cross-sectional

Vending data from facilities with a HVC at either T1 or T2 were compared with data from the remaining facilities with a conventional contract using a one-way ANOVA. A facility's data was only used once in the analysis and HVC data taken from the period first reported. Data was classified as missing if staff representatives were unsure of contract health stipulations at T1 and T2.

#### 2.5.2 Longitudinal

Longitudinal analyses examined changes in availability of healthier vending products over time and whether this differed by condition. There were four comparison conditions including: 1) facilities that reported a conventional contract or no contract at both T1 and T2, 2) facilities that reported a HVC at both T1 and T2, 3) facilities that reported a conventional contract or no contract at T1 and a HVC at T2, and 4) facilities that reported unknown at T1 and a HVC at T2. The number of facilities in each condition for beverage and snack vending machines is reported in Table 1.

**Table 1:** Number of facilities in each comparison condition (category of vending machine contract change) from T1 to T2 in beverage and snack vending machines used for longitudinal analysis.

Contract condition		Vending type	
T1	T2	Beverage (n)	Snack (n)
Conventional/no contract	Conventional/no contract	34	26
HVC	HVC	5	6
Unknown	HVC	5	3
Conventional/no contract	HVC	2	2

Six Repeated Measures Mixed ANOVAs were conducted to assess whether there were time and time by condition effects for percentage of DNS, SS or SM products across both beverage and snack vending machines. Assumptions of independence of observations and normalcy were met. To account for the assumption of sphericity, the Greenhouse-Geisser condition was used. Multiple post-hoc comparisons were performed using a Bonferroni correction to explore the significant effect of contract type.

## 3 Results

### 3.1 Cross-sectional

**Fig. 1:**
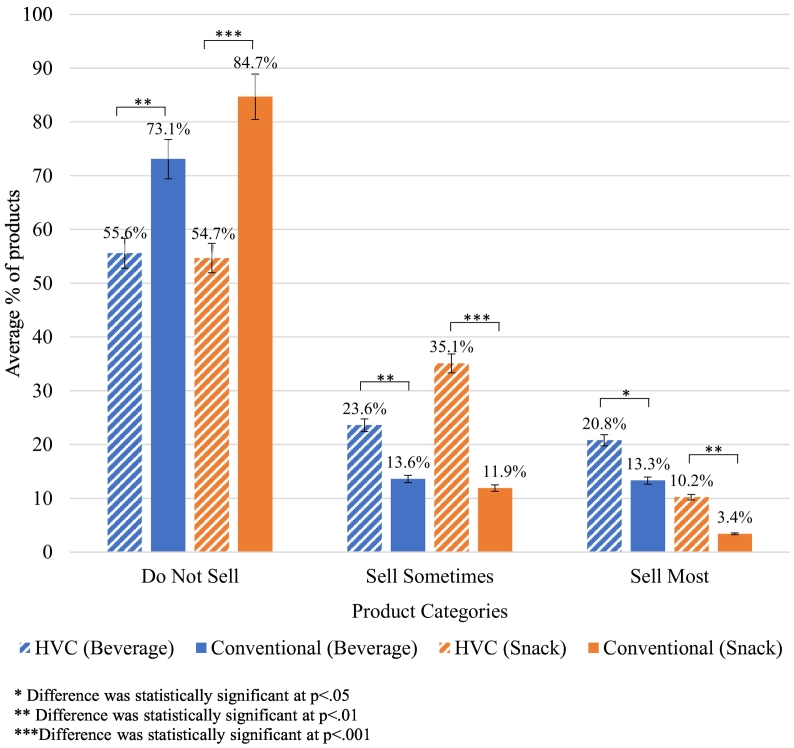
Cross-sectional comparison of the average percentage of Do Not Sell, Sell Sometimes and Sell Most products in snack and beverage vending by contract type (HVC or conventional).

The final sample size was 62 beverage and 43 snack vending contracts within the 46 participating facilities. Approximately a quarter of facilities had HVCs. Fig. 1 shows the average percentage of DNS, SS and SM vending machine products available in facilities with a HVC compared to facilities with a conventional contract. Over half of snack and beverage vending machine products at T1 were DNS irrespective of contract type. The availability of healthy products differed based on contract type for both snack and beverage vending machines, with higher percentages of SM and SS products and lower DNS products in facilities with HVCs compared to facilities with conventional contracts.

### 3.2 Longitudinal

**Fig. 2:**
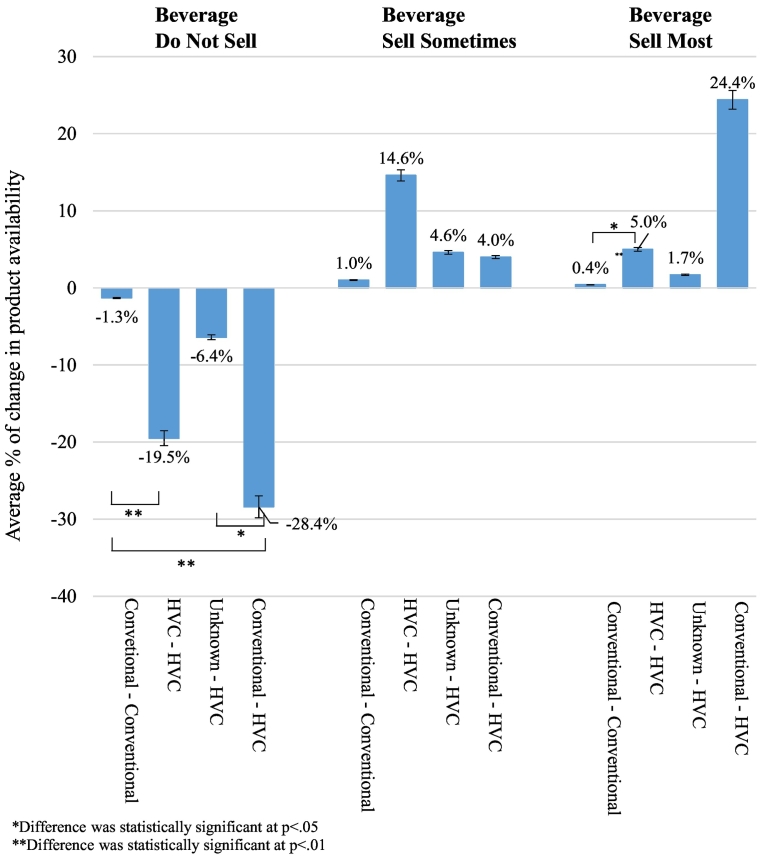
Longitudinal post-hoc comparison of the average % change in availability of Do Not Sell, Sell Sometimes and Sell Most products in beverage vending between T1 and T2 by contract change condition.

**Fig. 3:**
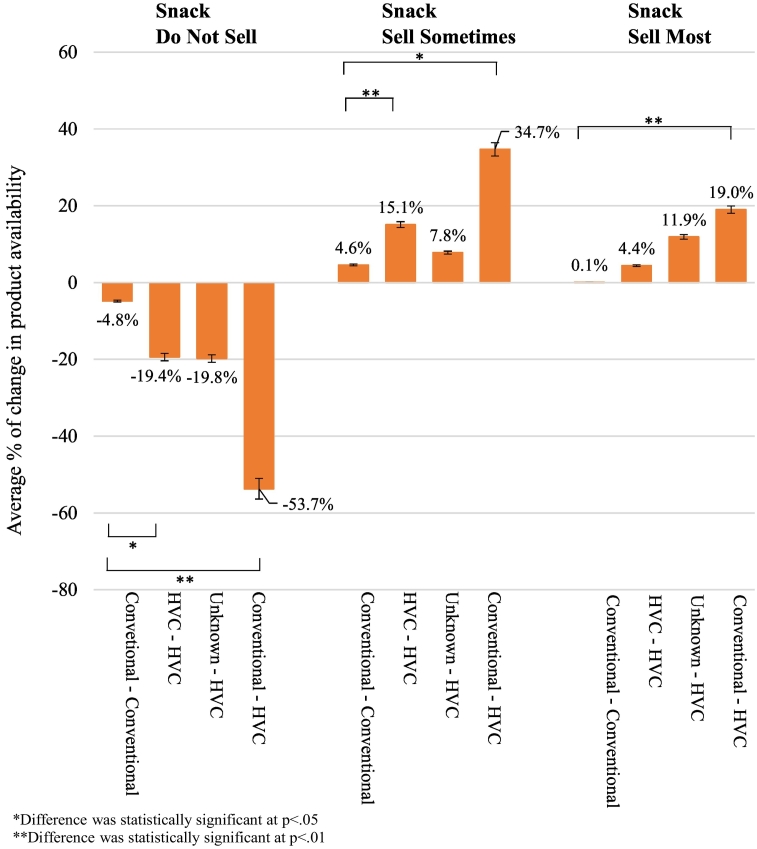
Longitudinal post-hoc comparison of the average % change in availability of Do Not Sell, Sell Sometimes and Sell Most products in snack vending between T1 and T2 by contract change condition.

Fifteen contracts were reported as ‘up for renewal’ during the 18-month EPL trial at T1. As displayed in Table 1, some contracts were changed between T1 and T2, with seven beverage and five snack vending contracts a HVC by T2. Fig. 2 (beverage) and Fig. 3 (snack) shows the percentage of change in the availability of SM, SS and DNS products between T1 and T2, for each condition detailed in Table 1.

#### 3.2.1 Beverage

For the DNS beverage condition, there was a significant main effect for time, *F* (1, 42) = 17.169, *p* < 0.001, partial eta^2^ = 0.290 and a significant interaction between time and contract type, *F* (3,42) = 4.491, *p* = 0.008, partial eta^2^ = 0.243. For the SS beverage condition, there was a significant main effect for time, *F* (1, 42) = 4.192, *p* = 0.047, partial eta^2^ = 0.091 but no significant interaction between time and contract type, *F* (3,42) = 1.801, *p* = 0.162, partial eta^2^ = 0.114. For the SM beverage condition, a significant main effect for time, *F* (1, 42) = 20.430, *p* < 0.001, partial eta^2^ = 0.327 and a significant interaction between time and contract type, *F* (3,42) = 7.358, *p* < 0.001, partial eta^2^ = 0.345. The differences in the percentage of DNS and SM beverages between T1 and T2 were not consistent across all categories of contract change.

The post-hoc testing (see Fig. 2) showed that the percentage of change between T1 and T2 for facilities with consistent HVCs was significantly different compared to those facilities with consistent conventional contracts The change over time was also significantly different for DNS products only, between facilities that changed from a conventional contract to a HVC and facilities with consistent conventional contracts. Changes also differed significantly over time for DNS products only, between facilities that changed from reported unknown health stipulations to a HVC and facilities that changed from a conventional to a HVC.

#### 3.2.2 Snack

For the DNS snack condition, there was a significant main effect for time, *F* (1, 33) = 32.117, *p* < 0.001, partial eta^2^ = 0.493 and a significant interaction between time and contract type, *F* (3, 33) = 6.185, *p* = 0.002, partial eta^2^ = 0.360. Similarly, for the SS snack condition, a significant main effect for time, *F* (1, 33) = 20.539, *p* < 0.001, partial eta^2^ = 0.384 and a significant interaction between time and contract type, *F* (3, 33) = 3.717, *p* = 0.021, partial eta^2^ = 0.253 was found and for the SM snack condition, also a significant main effect for time, *F* (1, 33) = 22.224, *p* < 0.001, partial eta^2^ = 0.402 and a significant interaction between time and contract type, *F* (3, 33) = 5.940, *p* = 0.002, partial eta^2^ = 0.351. This indicates that there was a difference in the percentage of all product categories between T1 and T2; like beverages however, this difference was not consistent across all categories of contract change.

The post-hoc testing (see Fig. 3) showed significantly different change between T1 and T2 for facilities with consistent HVCs for DNS and SS products only, compared to facilities with consistent conventional contracts. Change also differed significantly over time for all product types between facilities that changed to HVC compared with facilities with consistent conventional contracts.

## 4 Discussion

Current public health goals to promote healthy eating among children necessitate improving the food environments in the settings where children spend their time (World Health Organization, 2016). This includes Canadian publicly funded recreation and sport facilities (Thomas and Irwin, 2010; Naylor et al., 2010; Olstad et al., 2011a) where our data and previous evidence(Thomas and Irwin, 2010; Naylor et al., 2010; Chaumette et al., 2009; Olstad et al., 2019; Olstad et al., 2013; Olstad et al., 2015) indicate that less healthy foods and beverages remain the predominant option.

Vending machine contracts with health stipulations (HVCs) may be an effective policy tool (Olstad et al., 2013; Naylor et al., 2015; Naylor et al., 2010b; Brooks et al., 2017; Boelsen-Robinson et al., 2017; Lillehoj et al., 2015; Mason, 2014; van Hulst et al., 2013; Bell et al., 2013) to improve this situation, and may also be important for the implementation of broader food and beverage policies (Olstad et al., 2011a; Olstad et al., 2013; Naylor et al., 2015; Naylor et al., 2010b; Boelsen-Robinson et al., 2017; Bell et al., 2013). Few studies have specifically explored the impact of contracts as an intervention option to improve the food environment in Canadian publicly funded recreation and sport facilities. Thus, this study offers a novel exploration of the impact of HVCs in supporting the availability of healthier vending machine products in this setting.

We used both a cross-sectional and longitudinal analysis to explore whether nutritional quality of products differed in facilities with HVCs or that changed over to HVC between T1 and T2. Cross-sectional findings showed a greater availability of healthier vending machine products in facilities with a HVC at either time period. The longitudinal analysis supported the cross-sectional results and illuminated a plausible causal pathway. Vending product quality increased positively over time in facilities that had a consistent HVC (at T1 and T2) or introduced a HVC between T1 and T2 compared to those facilities with conventional contracts. We discuss our findings in the context of the literature and strengths and limitations of the research following.

### 4.1 The impact of healthy vending contracts

Although the majority of facilities in this study reported having a vending contract, only a small number of the contracts met the HVC criteria and only a few facilities added health stipulations to their contracts over the 18-month EPL intervention period. This is not surprising considering that some of the facilities were EPL comparison sites that were asked not to make changes and evidence indicates that changing contracts may take longer than the study time frame depending on contract renewal dates (Naylor et al., 2015).

Cross-sectional analyses showed that facilities with HVCs had better availability of healthy beverage and snack vending products. Further, longitudinal findings showed that facilities that had a consistent HVC or changed to a HVC had significant improvements in the nutritional quality of vending products over time compared to those that did not. Our findings reinforce the positive association between HVCs and availability of healthier products found in other settings (Brooks et al., 2017; Boelsen-Robinson et al., 2017; Mason, 2014; Bell et al., 2013), as well as previous research where recreation facilities with a HVC appeared to have a substantially greater change in healthy vending products (Naylor et al., 2010b) or were more likely to adopt provincial guidelines (Olstad et al., 2011a).

The most substantial improvements in the nutritional quality of vending machine products between T1 and T2 were the reduced availability of the least healthy (DNS) snack and beverage products. This corresponds with research in the health services setting linking a HVC with greater reductions in availability of unhealthy products (Boelsen-Robinson et al., 2017), and suggests that contract health stipulations may have a greater influence on decreasing unhealthy options as opposed to increasing healthier ones. A lesser effect on the healthiest products may be due to numerous barriers including: increased complexity, difficulty sourcing, limited convenience, loss of revenue, lack of consumer demand, and insufficient resources (Olstad et al., 2011a; Olstad et al., 2013; Naylor et al., 2015; Olstad et al., 2011b; Boelsen-Robinson et al., 2017; van Hulst et al., 2013; Grech and Allman-Farinelli, 2015). Despite this, decreasing unhealthy products remains important, as it has contributed to improved overall nutritional quality in vending machines (Boelsen-Robinson et al., 2017; Grech and Allman-Farinelli, 2015) and increased the likelihood of patrons' purchasing a healthier choice (Olstad et al., 2015b). Furthermore social norms are anticipated to change parallel to policy related changes in access (Ashe et al., 2007; Schwartz et al., 2017). Thus, it may be sufficient to focus initially on reducing availability of unhealthy products while stakeholder acceptance increases and barriers to supplying healthy products are addressed.

### 4.2 Contract renewals

An inevitable limitation of HVCs as a form of policy is their temporary nature which does not ensure sustainability and could be especially problematic without sufficient time to actualize positive change. This was one of the issues cited following discontinuation of Denmark's enforced tax paid on saturated fat content in food after 1 year which negated time for “incremental health effects to accumulate and become practically significant” (Sisnowski et al., 2015). It may also be problematic when no underpinning facility or state-level policy is present whereby changes in staffing could result in a loss of institutional memory and consequently regression to unhealthy contracting practices.

Nevertheless, the impermanence of vending contracts also allows for renewals which have previously been used to improve the health of food and beverages in public settings (Boelsen-Robinson et al., 2017; Bell et al., 2013) including recreation facilities.(Naylor et al., 2010; Olstad et al., 2011a; Olstad et al., 2013; Naylor et al., 2015). In this study, facilities that changed their status from conventional contract to HVC significantly improved the healthfulness of vending. This suggests that adding health stipulations to a vending contract during renewal may be both feasible and effective within a short period of time. Moreover, it appeared that positive changes occurred in facilities with an established HVC, signifying that the impact may strengthen with time. The large number of facilities at T2 with conventional or no contract shows the potential for enacting HVCs in Canadian publicly funded recreation and sport facilities when contracts come up for renewal.

### 4.3 Study limitations and strengths

Caution should be used when interpreting the findings of this study based on the limitations of its real world non-equivalent quasi-experimental comparison design. These designs do not control for a number of threats to validity including history, maturation or selection bias and interaction among these. It is also likely that only those facilities interested in changing their food environment participated which introduces issues of sampling bias. However, it should be noted that even among facilities that volunteered, having a HVC or changing to a HVC was not frequent. Also mentionable is that this analysis only addresses the outcomes of a HVC, and not factors that influenced adoption and implementation. A related limitation is the small number of facilities with HVCs, which increased the likelihood that a true difference may have been missed (Fink, 2013). Further, the external validity of our findings are limited due to the small sample size of facilities (<3%) used compared to the approximately 1924 community centers (excluding individual ice rinks, swimming pools etc.) found within the four participating Canadian provinces (Statistics Canada, n.d.). The use of self-report from facility representatives regarding vending contract status was another limitation, in particular social desirability bias. This could have been exacerbated when facility staff who had not completed the emailed survey were followed up with and interviewed verbatim over the phone. Interviews allow for clarifications so it may be that their data was more accurate than that of survey respondents.

Strengths of this study include the ‘real world context’ in which it was implemented, it's use of both cross-sectional and longitudinal analysis to explore the influence of HVCs, as well as the use of data from multiple provinces which supports the generalizability of our findings across jurisdictions.

## 5 Conclusion

Our findings add to a growing literature base and establish a causal pathway between HVCs and the availability of healthier food and beverage vending products specifically in recreation and sport facilities, which may over time influence social norms. This research supports the importance of facility-level policy in the form of HVCs to achieving guideline implementation in Canadian and other jurisdictions. A substantive finding was the relatively short-time frame to achieve change in facilities that either maintained a HVC or changed to a HVC; a facility-level action. These are notable findings considering the limited resources and barriers governments face in the food and beverage policy field (Olstad et al., 2013; Crammond et al., 2013; MacRae, 2011; Rose and Cray, 2010).

Unfortunately, it is also important to note how far off the ‘ideal’ facilities remained. Vending audits indicated no facilities met the BC provincial guidelines (Province of British Columbia, 2014) of 50% SM and 50% SS products, nor were any near achieving these standards. Moreover, specific nutrition standards or contract monitoring were not commonly articulated in contracts. A continued emphasis on strengthening food and beverage policies in this setting is fitting (Olstad et al., 2019; Olstad et al., 2011a; Olstad et al., 2013). Future research examining the impact of food and beverage policies on other aspects of food environments within publicly funded recreation and sport facilities (i.e., cafes, restaurants and concessions) is needed.

## Authorship

SFLK, RH, LCM, DLO, KR, PJN designed the overall Eat, Play, Live study, obtained funding and oversaw ongoing implementation; RP, SC and DT collected and entered all of the data; CL and PJN formulated the research questions and designed the sub-study; CL, DT and TM analysed the data; CL drafted the manuscript; and all authors revised the manuscript.
